# Osteoporosis and Bone Fragility in Children: Diagnostic and Treatment Strategies

**DOI:** 10.3390/jcm13164951

**Published:** 2024-08-22

**Authors:** Giuseppe Cannalire, Giacomo Biasucci, Lorenzo Bertolini, Viviana Patianna, Maddalena Petraroli, Simone Pilloni, Susanna Esposito, Maria Elisabeth Street

**Affiliations:** 1 Paediatrics and Neonatology Unit, University of Parma, Guglielmo da Saliceto Hospital, 43121 Piacenza, Italy; g.cannalire@ausl.pc.it; 2 Department of Medicine and Surgery, University of Parma, 43126 Parma, Italy; simone.pilloni@unipr.it (S.P.); mariaelisabeth.street@unipr.it (M.E.S.); 3 Unit of Paediatric Radiology, University Hospital of Parma, 43126 Parma, Italy; 4 Unit of Paediatrics, Department of Mother and Child, University Hospital of Parma, 43126 Parma, Italy

**Keywords:** osteoporosis in children, bone fractures, bone mineral density, bisphosphonates, denosumab

## Abstract

The incidence of osteoporosis in children is increasing because of the increased survival rate of children with chronic diseases and the increased use of bone-damaging drugs. As childhood bone fragility has several etiologies, its management requires a thorough evaluation of all potentially contributing pathogenetic mechanisms. This review focuses on the main causes of primary and secondary osteoporosis and on the benefits and limits of the different radiological methods currently used in clinical practice for the study of bone quality. The therapeutic and preventive strategies currently available and the most novel diagnostic and treatment strategies are also presented. Optimal management of underlying systemic conditions is key for the treatment of bone fragility in childhood. DXA still represents the gold standard for the radiologic evaluation of bone health in children, although other imaging techniques such as computed tomography and ultrasound evaluations, as well as REMS, are increasingly studied and used. Bisphosphonate therapy is the gold standard for pharmacological treatment in both primary and secondary pediatric osteoporosis. Evidence and experience are building up relative to the use of monoclonal antibodies such as denosumab in cases of poor response to bisphosphonates in specific conditions such as osteogenesis imperfecta, juvenile Paget’s disease and in some cases of secondary osteoporosis. Lifestyle interventions including adequate nutrition with adequate calcium and vitamin D intake, as well as physical activity, are recommended for prevention.

## 1. Introduction

Bone health in children is of utmost importance, as it influences global development and quality of life throughout life. Assessment of bone health may be difficult because of congenital or acquired factors, including primary skeletal disorders or secondary causes of reduced bone accrual such as alterations in pubertal development, endocrinopathies, altered weight bearing and pharmacological therapies. Bone metabolism is regulated by environmental factors that modulate bone formation and resorption of older bone. The anabolic and catabolic actions in bone are balanced thanks to the tight regulation of cells that form bone (osteoblasts) and resorb bone (osteoclasts) [1]. Osteoblasts and osteoclasts are regulated also by hormones; in particular, testosterone and growth hormone stimulate the anabolic activity of osteoblasts, while cortisol has an inhibitory effect. Bone resorption is favored by thyroid hormones, parathyroid hormones and inflammatory cytokines, while the osteoclast function is inhibited by estrogens. Calcium and phosphorus deficiencies lead to a poorly mineralized bone matrix [2].

The current definition of pediatric osteoporosis refers to a bone mineral density (BMD) Z score ≤ −2 with respect to the reference population and is associated with a clinically significant history of fractures, i.e., two or more long bone fractures by 10 years of age, three or more fractures of long bones at any time up to the age of 19 years of age or one or more vertebral compression fractures occurring without high-energy trauma or local disease, regardless of BMD Z score [3].

The standards of bone health and mineral density are set mainly by data provided by Dual-energy X-ray Absorptiometry (DXA), which allows for the evaluation of bone mineral content, non-bone lean mass and fat mass and to assess the amount of visceral adipose tissue. The World Health Organization’s T-score definition of osteoporosis allows for interpretation of the results of DXA BMD, prediction of the risk of fractures and evaluation of therapeutic follow-up in patients undergoing anti-fracture therapy but is applicable to the adult population only [4]. Osteoporosis in children can be caused by genetic and acquired bone pathologies, which may potentially lead to fractures during childhood. Primary osteoporosis is usually caused by underlying genetic disorders. Secondary osteoporosis develops because of the effects of a chronic illness or related treatments [1,3,4]. The therapeutic approach to osteoporosis includes several treatment options. Bisphosphonates (BPs) are currently the most used drugs. In recent years, some novel and promising therapies that act on the signaling pathways involved in the formation and remodeling of bones have started to be studied and used in childhood [1,5].

This review presents a comprehensive discussion of the pathogenesis of osteoporosis in children, with specific reference to the primary and secondary causes of this condition, with the aim of describing the state of the art in osteoporosis management in children and the future directions for the diagnosis and treatment of this condition. The diagnostic evaluation of bone health in children is also discussed, focusing especially on imaging techniques. Interestingly, new diagnostic approaches, such as Quantitative Computed Tomography (QCT), Quantitative Ultrasound (QUS) and Radiofrequency Echographic Multi Spectrometry (REMS), have been increasingly studied and used for this purpose in recent years, although DXA remains the gold standard for diagnosis. Finally, preventive and treatment strategies are addressed, highlighting the importance of an adequate diet and specific supplementations in children. Concerning therapy, the value of bisphosphonates and novel therapies such as denosumab is discussed.

## 2. Methods

This narrative review presents a concise but broad overview of the risk factors for bone fragility, its diagnostic workup and the therapeutic approach in pediatric patients with osteoporosis.

### Literature Search Strategy and Selection Criteria

A literature search was performed using specific research strings in the Pubmed and MeSH databases comprising specific keywords. Original articles, including animal and in vitro studies, reviews and epidemiological studies, were selected, focusing initially on those published in the last 10 years. The authors separately screened the included papers found through the literature search by title and abstract. For specific topics, when the literature was scarce, we included even older publications. The following keywords were used for the search: osteoporosis, bone fragility, bone health, primary osteoporosis, secondary osteoporosis, osteogenesis imperfecta, diagnosis, DXA, bone density, treatment, bisphosphonates, denosumab, calcium, phosphorus, parathyroid glands, PTH and vitamin D.

## 3. Discussion

### 3.1. Primary Osteoporosis

Primary osteoporosis includes genetic forms of skeletal fragility defined as Osteogenesis Imperfecta (OI). These are a group of complex and heterogeneous conditions characterized by an elevated risk of bone fractures, showing different severity; most are associated with collagen type I biosynthesis deficiency, which is the largest component of the extracellular matrix in bones, skin and tendons and is secreted by osteoblasts, skin fibroblasts and tenocytes. The prevalence of OI ranges from 1/10.000 to 1/20.000 [6,7]. Most patients (about 90%) affected by OI have genetic defects in the *COL1A1* or *COL1A2* genes. OI patients present fractures without trauma or with low-impact trauma, without the presence of other bone disorders. More common extraskeletal phenotypes are short stature or stature below mid-parental height, associated with bone deformity, blue sclerae, ligamentous laxity or other signs of connective tissue anomalies [7]. Different classifications in OI have been speculated upon according to clinical features, genetic findings or pathogenetic mechanisms [8]. Starting from Sillence’s initial classification, Van Dijrk et al. classified the forms of OI by grouping them into five types by age of onset and severity of the clinical phenotype, progressive deformation, histologic characteristics and inheritance of different genetic mutations [8,9], with this classification recently modified by Unger et al. [6] (Table 1). Radiological features may be suggestive of OI change over time. Fractures often occur in the long bones and, sometimes, the ribs and skull, whereas metaphyseal fractures can be seen in a very small number of children with OI. “Codfish” vertebrae are more commonly seen in adults due to spinal compression fractures. Osteoporosis is often shown by DXA, although since DXA measures mineral content rather than collagen, BMD may be normal, especially in individuals with OI type I [10,11,12,13].

Another cause of primary osteoporosis in childhood is classical homocystinuria, an autosomal recessive disease characterized by elevated plasma homocysteine and methionine levels due to reduced activity of the cystathionine beta-synthetase enzyme, the enzyme that catalyzes the conversion of homocysteine to cystathionine. The effect of homocystinuria on bone health is due to high homocysteine levels, which increase osteoclast activity; induce apoptosis in bone marrow stromal cells, osteocytes and osteoblasts and inhibit the differentiation of osteoblasts [14,15,16].

Cleido-cranial dysostosis is a rare genetic disease with autosomal dominant inheritance characterized by defects in ossification, delayed bone and dental development, and stomatognathic and craniofacial alterations. It is caused by mutations in the *RUNX2* gene, which is responsible for osteoblast differentiation. Patients present with proportionate dwarfism and the distinctive feature of hypoplastic or absent clavicles [8].

A very rare genetic condition associated with primary osteoporosis is Osteoporosis Pseudoglioma Syndrome (OPPG), which is caused by a loss-of-function mutation in the low-density lipoprotein receptor-related protein 5 (LRP5) gene, which encodes for Wnt co-receptor LRPR5. The clinical characteristics of patients affected by this disease can include congenital or very early-onset visual loss (the most typical feature), muscular hypotonia, ligamentous laxity, mental retardation, obesity and early-onset severe osteoporosis [17,18,19].

Several conditions causing impairment in the development of connective tissue or bone can be associated with a reduction in BMD and associated increased fracture risk. One example is Marfan Syndrome (MS). MS is a systemic disorder of connective tissue caused by mutations in extracellular matrix protein fibrillin 1. MS is a multisystemic disorder, with the most typical manifestations involving the cardiovascular, skeletal and ocular systems. The skeletal characteristics in MS include overgrowth of long bones, causing classical disharmonic stature; protrusion of the acetabula; scoliosis; and decreased BMD [20].

Ehlers–Danlos Syndrome (EDS) is another condition in which a reduction in BMD can be observed. The spectrum of EDS includes several connective tissue disorders characterized by joint hypermobility, stretchy and easily bruised skin and fragility in various tissues. EDS is a genetically heterogeneous group of disorders with as many as 19 different disease-associated genes [21].

Decreased BMD can also be observed in hypophosphatasia, a condition characterized by an autosomal dominant or autosomal recessive inborn error of metabolism with a great range of severity. This condition is caused by loss-of-function mutations in the gene that encodes for the tissue-nonspecific isoenzyme of alkaline phosphatase [22].

Finally, idiopathic juvenile osteoporosis may be diagnosed by excluding all other possible causes, as the underlying pathophysiology remains unclear. However, there is an increasing awareness that some children previously diagnosed with idiopathic juvenile osteoporosis have pathogenic variations in bone signaling pathways [23]. Idiopathic juvenile osteoporosis affects both genders equally and typically presents in a subtle way, often in pre-pubertal children, with symptoms such as back pain, hip and/or lower-limb pain, vertebral fractures, long bone fractures and problems walking [24].

### 3.2. Secondary Osteoporosis

Reduced BMD in children can be a consequence of several conditions, including diseases and side effects of drugs used for long-term therapies. Diseases include malabsorption disorders (e.g., gluten intolerance, inflammatory bowel diseases, cystic fibrosis, etc.), conditions characterized by reduced bone stress (cerebral palsy, muscular dystrophies, hypotonia, spina bifida, etc.), endocrine disorders (hypogonadism, hypercortisolemia, growth hormone deficiency, etc.), and oncologic and hematologic diseases (e.g., leukemia, beta-thalassemia, etc.). Drugs used for long-term therapies potentially related to detrimental effects on children’s bone health mainly include steroids and proton-pump inhibitors [25,26,27,28,29,30].

Overall, endocrinological disorders are characterized by changes in the levels of hormones that may impact bone health and favor secondary osteoporosis in children and adolescents. Sex hormones, in particular, have a crucial role in the development of the skeleton, among which estrogen and progesterone are key. Estrogen is one of the hormones responsible for the pubertal growth spurt, which is associated with a doubling of the skeletal mass and is also crucial for peak bone mass achievement in both males and females. Testosterone exerts an additional action by stimulating the apposition of the periosteum, thereby accounting for the larger size and increased thickness of the cortex of the male skeleton [31,32,33]. Thyroid disorders may have severe effects on skeletal development in children and adolescents. Indeed, hypothyroidism in children delays skeletal development and mineralization, and replacement with thyroid hormones is essential to guarantee bone maturation and catch-up growth; it is, therefore, crucial to maintain the euthyroid status in childhood and adolescence in order to establish an adequate peak bone mass. Not only hypothyroidism but also untreated hyperthyroidism is associated with accelerated bone remodeling, reduced BMD and increases in the fracture rate [34].

Other childhood endocrinological disorders possibly associated with reduced BMD include hypercortisolemia, hyperparathyroidism (also induced secondarily by hypocalcemia and vitamin D deficiency), growth hormone deficiency and resistance and diabetes [35].

Glucocorticoids inhibit the activation of mature osteoblasts and interfere with the ability of the precursors of osteoblasts to adhere to the matrix. They also increase bone resorption by inhibiting gonadotropin secretion, with subsequent drops in estrogen and androgen levels, which cause accelerated bone mass loss in adolescents [30,36,37].

Methotrexate, a folic acid antagonist, and calcineurin phosphatase inhibitors such as tacrolimus stimulate osteoclast activity by interfering with interleukin 1 and 6 synthesis [38].

Children diagnosed with Duchenne muscular dystrophy and other myopathies that require high-dose steroid therapy have an increased risk of developing osteoporosis that is also related to the reduced mechanical stimulation of bone and reduced weight bearing of bones because of reduced mobility/immobility. Osteoporosis in these subjects can frequently present with asymptomatic vertebral fractures (VFs) or fractures in long bones [36,37].

Antiepileptic drugs such as carbamazepine activate the expression of cytochrome P450 CYP24A1, accelerating the catabolism of active vitamin D metabolites, determining a state of vitamin D deficiency. In addition, valproic acid increases osteoclast activity, favoring a low BMD [31,39,40]. Progestins suppress the secretion of pituitary gonadotropins and ovarian production of estradiol and estrone, all of which are essential for bone metabolism.

Gonadotropin-releasing hormone agonists administered for the treatment of precocious puberty suppress the production of gonadotropins and cause a condition of hypogonadotropic hypogonadism associated with bone loss; however, this condition is reversible upon discontinuation of therapy.

Aromatase inhibitors increase bone resorption and accelerate bone loss by reducing 17β-estradiol levels. [32].

Oncological disorders are listed among the conditions that are associated with a reduction in BMD and an increased risk of fractures in children and adolescents. Acute lymphoblastic leukemia is characterized by a consistent skeletal morbidity. Derangement in bone metabolism is observed in this disease at diagnosis (as many as 75% of children with acute lymphoblastic leukemia have skeletal abnormalities at diagnosis), during treatment and after treatment. In this case, bone fragility seems to be linked to increased bone resorption by osteoclasts, which is induced by the cytokines directly released by the cancer cells [29]. Studies have shown that most bone morbidity (including long-bone and vertebral fractures) presents within the first two years after the start of therapy. In children who receive therapy for cancer, the factors that are associated with poor bone health include chemotherapies having toxic effects on bone, treatment with glucocorticoids, poor nutritional status, vitamin D insufficiency and reductions in muscle mass, all contributing to secondary osteoporosis, long-bone and vertebral fractures and osteonecrosis [30].

Finally, we know that radiation therapy has direct negative effects on osteoblasts [2].

Besides the above-mentioned conditions, anorexia nervosa may also lead to bone metabolism disruption because of severe undernutrition and hypothalamic dysfunction in both male and female adolescents. Poor bone health is a consequence of changes in body composition and is characterized by reduced bone turnover, bone cortical thickness and low BMD. Moreover, anorexia nervosa is associated with changes in hormones with an impact on bone quality, including anorexigenic hormones such as leptin, peptide YY and GLP1 [33].

With regard to obesity, the main negative effects of this condition on bone tissue are mediated by the increased differentiation of mesenchymal stem cells towards adipocytes at the expense of osteoblasts, reduced levels of physical activity with increased activity of osteoclasts, reduced intestinal calcium absorption and low-grade chronic inflammation. In addition, it is important to underline that unhealthy lifestyle choices such as smoking and alcohol consumption can negatively affect bone health. This must be considered in adolescents and young adults [41]. Furthermore, the impact of systemic inflammation caused by air pollution may have long-term detrimental effects on bones in children, increasing the risk of osteoporosis [42].

Exposure to endocrine-disrupting chemicals (EDCs) has also been associated with changes in the regulation of bone formation and remodeling, particularly in the most critical periods of development such as fetal life and puberty. These effects are the consequences of increased bone resorption and reduced bone deposition, which may also affect bone mass accrual during adolescence. The effect of EDCs on bone health has been shown in several in vitro experiments. For instance, xenoestrogens exert a negative effect on bone by binding to α and β estrogen receptors on osteoblasts and osteoclasts, thereby modifying the equilibrium between bone resorption and formation. Studies in rodents have also shown the effects of EDCs on bone development and structure. Human studies have also suggested that prenatal exposure to EDCs can negatively influence bone health in fetuses directly or through the dysregulation of placental functions. Considering single EDCs, maternal serum bisphenol S levels have been clearly associated with lower bone mineral density and bone mineral content in offspring at 10 years of age [43,44,45].

## 4. Evaluation of Bone Health and Bone Density in Children

### 4.1. Physical Examination and Biochemical Exams

The evaluation of bone health in children must always start with a thorough collection of medical history and physical examination. Medical history must be addressed to identify events related to bone fragility (e.g., fractures and their characteristics, back pain, etc.) and risk factors that may predispose to bone fragility, including comorbidities, use of drugs, non-physiological pubertal development, etc. Family history is always of importance, especially considering the risk for genetic diseases. The findings that emerge address the following steps in the diagnostic workup. The physical examination of a child with possible bone fragility needs to focus on the general assessment of the child but also on factors that may disclose a specific disorder. For instance, together with the general anthropometric parameters, teeth, eye, joint, skin and spine features must be carefully assessed. It must also be kept in mind that no single blood test can confirm the diagnosis of osteoporosis, but a general evaluation of bone metabolism is required. Blood tests must include calcium, phosphate, alkaline phosphatase, 25-hydroxy vitamin D, parathormone, magnesium, creatinine, albumin and gamma glutamyl transferase. The initial tests help to exclude disorders characterized by reduced bone mineralization, such as rickets. When these disorders are ruled out, systemic conditions that may initially present with bone fractures should be included in the diagnostic process. Since the exclusion of secondary causes of osteoporosis is fundamental when evaluating a child with reduced bone density [46], the assessment of urinary calcium, creatinine and phosphate; celiac disease screening; and thyroid evaluation should also be performed. Erythrocyte sedimentation rate measurement is key if systemic inflammatory conditions are considered in the differential diagnosis. In the absence of a clear cause of osteoporosis or in the presence of a suspected genetic disorder, genetic tests should be performed [1].

**Figure 1:**
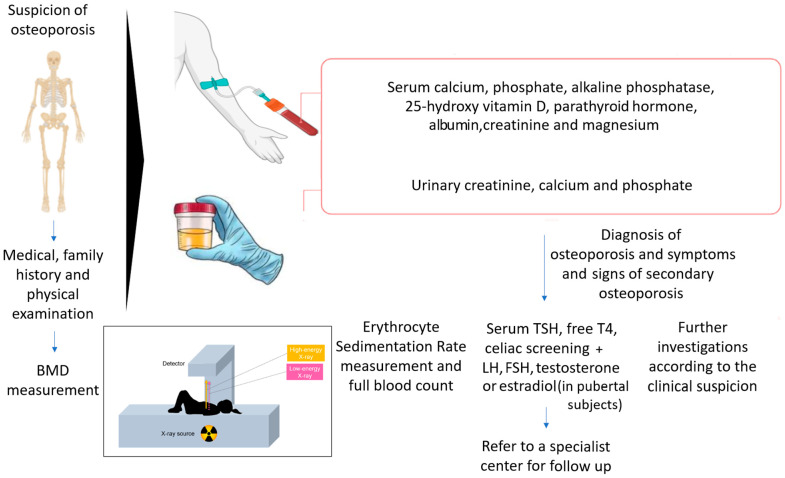
Diagnostic workup for children with suspicion of osteoporosis.

The overall diagnostic workup in a child suspected of osteoporosis is summarized in Figure 1.

Finally, Bone Turnover Markers (BTMs) deserve a mention. When measured in serum, these markers are considered surrogates of bone formation and bone resorption. The most important marker of bone formation is serum procollagen type I N propeptide (s-PINP), while the most relevant marker of bone resorption is serum C-terminal telopeptide of type I collagen (s-CTX) [47]. The use of BMTs in the diagnosis and monitoring of osteoporosis in children is, however, controversial. This is understandable if we consider that bone turnover is physiologically increased in childhood, posing many challenges in the development of reference standards for normal values in this period of life.

### 4.2. Skeletal Imaging Techniques

Skeletal imaging techniques have increasingly become a key tool in clinical practice to measure and evaluate bone mineral density. Overall, the concept of “bone health” describes the properties of skeletal tissue, and it can be assessed by evaluating bone resistance to fractures, known as “bone strength”.

The ability to evaluate bone strength and measure bone mineral density has evolved significantly in recent decades. The main purpose of these evaluations is to identify patients with a high risk of fractures following low-intensity trauma, potentially due to bone fragility, as well as to guide therapeutic decisions and monitor therapeutic effects [3,48].

DXA represents the gold standard for evaluating bone density parameters in children. In DXA, an X-ray beam with two distinct energy spikes passes through bones and soft tissues with different absorption rates and is collected by a detector. Based on the reduction in beam intensity, bone mineral content is extrapolated mathematically by subtracting the soft tissue component. In clinical practice, rather than BMD values, the reference parameter is the Z score (number of standard deviations with respect to a reference population of the same age). The lumbar spine, radius, hip and total body can be tested. According to the recommendations of the International Society for Clinical Densitometry (ISCD) [3,49] measurement sites for DXA in most children include the posterior–anterior lumbar spine measurement and the total body less head measurement. Depending on the clinical need, other sites may also be useful. The hip is not considered an adequate site in growing children because of its high variability. In addition to bone density, these scores allow for an accurate measurement of body composition, e.g., the quantitative evaluation of muscle and adipose tissue [50].

The ISCD recommends that the minimum interval between follow-up DXA scans should be at least 6 to 12 months and, with regard to the correct terminology for reporting in children, suggests that terms such as “osteopenia” and “osteoporosis” (in the absence of a significant fracture history) should not appear and should be substituted by expressions such as “low bone mass” or “low mineral density”, in accordance with the recognized diagnostic criteria for osteoporosis in children and adolescents. The main limitations of this method in childhood are represented by the lack of solid reference databases (especially in children under 4 years of age), the lack of significant outcomes linked to densitometric measurements, and inaccuracies and artefacts due to changes in size and body composition in relation to growth [51,52]. In children, DXA BMD measurements are, in fact, influenced by height. Although DXA measurements of areal BMD incorporate bone size, bone size adjustment is incomplete because the two-dimensional image does not incorporate bone depth; therefore, smaller bones with comparable volumetric BMD appear to have lower areal BMD. To reduce the confounding effect of short stature on spine bone density, bone mineral apparent density and height-for-age Z score are used and recommended [51].

Computed tomography techniques for the evaluation of bone health in children include QCT, peripheral QCT (pQCT) and vertebral QCT (vQCT). These techniques allow for the attainment of information on bone geometry (which is impossible to obtain with DEXA) by assessing cortical and trabecular bone separately [1]. QCT has important distinctive characteristics because it allows for a three-dimensional study of the bone. It can measure volumetric bone mineral density (expressed in g/cm^3^ and defined as vBMD [51]), independent of bone size. It is also possible to evaluate the structure and geometry of the segments under examination (e.g., the axial sectional area and the periosteal and endosteal circumference). Moreover, in next-generation scanners, radiation exposure is relatively low (0.59–1.09 mSv) [53,54,55]. In children, QCT studies are usually performed as pQCT, allowing for evaluation of the radius or the tibia or the femur separately. pQCT is able to evaluate multiple bone parameters, such as metaphysis and mid-shaft sites. High-Resolution-pQCT (HR-pQCT) is an even more sophisticated technique that can reduce the radiation dosage by evaluating small regions of the tibia and radius. HR-pQCT can provide information on total bone area, vBMD and specific characteristics of cortical and trabecular bone [56]. Recent studies have elaborated on bone density reference intervals for pQCT [57].

Magnetic resonance imaging (MRI) is not yet used routinely in clinical practice but provides volumetric measurements of the bone and, similarly to QCT, can evaluate cortical and trabecular components separately. However, the image acquisition time is quite long (approximately 20–30 min), and sedation is required in younger children. The most recent evolution in MRI is the imaging technique called microMRI, which allows a “virtual biopsy” with the resolution of single-bone trabeculae [52,58].

The use of QCT and MRI is reserved for isolated cases and is difficult to carry out on a large scale.

### 4.3. Ultrasound Evaluation of BMD

QUS scan evaluates bone in both quantitative and qualitative fashions; hence, it provides a measure of “bone quality” and is currently used as a global indicator of “bone strength”. The ultrasound equipment consists of two transducers, a transmitter and a receiver. The ultrasound wave produced by the transmitter passes through the bone segment under examination and is received by the second transducer [10]. The advantages of QUS for children include safety, ease of use and cost-effectiveness. Moreover, the exam takes just a few minutes to be performed, and the devices are portable, allowing for bedside evaluations. Another characteristic that makes QUS particularly applicable in children is the lack of radiation emitted by the technique. The measurements are usually taken at the level of the heel, proximal phalanges of the hand, tibia or radius [59]. Comparison between bone mineral density measured by QUS and DXA has showed conflicting results [60]; therefore, this technique is currently not applicable in clinical practice and has been seldom used because there are no reliable reference values and it cannot be used for the axial skeleton [61].

A new ultrasound approach for the diagnosis of osteoporosis directly applicable to both the femur and the lumbar spine has recently been introduced and clinically validated. This developed approach is called REMS, with data obtained by analyzing the raw and unfiltered ultrasound signals, i.e., so-called radiofrequency ultrasound signals obtained from lumbar vertebrae and the proximal femur. The main parameter provided by this technique is BMDUS, a diagnostic index expressed in grams/cm^2^, which has shown significant correlations with the corresponding BMD values and a high degree of concordance with data based on DXA; therefore, it is taken as the reference gold standard. The evaluation of native ultrasound signals provides the best information on the features of the examined tissues. In particular, bone health status is measured through the comparison of the analyzed signal spectra with derived reference spectral pathological and normal models from a specific database, which allows for classification of bone tissue as healthy, osteopenic or osteoporotic. The high sensitivity and specificity of REMS technology for the identification of osteoporosis and osteopenic patients have been demonstrated by multiple clinical studies. The REMS radiation-free approach could represent a valid instrument for population screening and clinical practice [43].

A list of the most relevant imaging modalities for the assessment of bone health in patients of pediatric age is presented in Table 2.

## 5. Treatment and Prevention

Drugs active on bone and some non-pharmacological therapies can increase bone mass, reducing the risk of fractures. As already described, lifestyle changes such as increased physical activity, reduced alcohol intake and smoking cessation are recommended prevention strategies for adolescent osteoporosis. Food supplementation with vitamin D and calcium and a varied and balanced diet with adequate protein; trace elements; and vitamins A, C and K are also highly recommended for bone health [2,4]. In children between 0 and 6 months and between 6 and 12 months, the optimal calcium intake is 200 and 260 mg/day, respectively. In children over 1 year of age, the adequate dose of calcium to be introduced through diet is 700 mg/day. In addition, all infants should receive 400 UI/day vitamin D supplementation throughout the first year of life, regardless of their feeding mode. Thereafter, children with a history of symptomatic vitamin D deficiency and those with risk factors that may reduce the synthesis or intake of vitamin D are candidates for supplementation. Finally, exposure of skin to sunlight on the hands, limbs and face for a minimum time of 6–8 min a day during the summer and half an hour a day during fall and winter is also of utmost importance [62]. Daily calcium and vitamin D requirements according to age are shown in Table 3 [63].

Bisphosphonates (BPs) inhibit bone resorption acting on osteoclasts, form chelates with calcium ions and bind to hydroxyapatite on the exterior part of the bone. BPs are indicated in cases of difficult treatment of the underlying comorbidity in secondary osteoporosis and in the case of sustained recurrent fractures, especially in monogenic forms of early-onset osteoporosis. Their effectiveness has been demonstrated for the treatment of Paget’s disease and bone marrow edema. Among the adverse effects, gastrointestinal symptoms are reported after oral administration, and flu-like symptoms may occur after intravenous injection. Generally, the side effects of BPs are minimal.

No case of osteonecrosis of the jaw has been reported in children with osteoporosis receiving BPs, but caution and avoidance of dental procedures when receiving intravenous BPs are recommended.

Kidney function should be checked before starting BPs, as they should be used with caution in patients with renal failure. Furthermore, esophagitis consequent to oral BP administration has been described [2]. A proportion of 50% of administered BPs is excreted by the kidneys, while the remaining 50% is absorbed by the skeleton [63].

The studies currently present in the literature do not provide a unanimous consensus on the choice of which BPs to use or dosage and duration of treatment. Intravenous administration is considered to provide a greater benefit to vertebral fractures. An international randomized, intravenous, placebo-controlled study on zoledronic acid published in 2021 confirmed intravenous administration of BPs as the most effective therapy for osteoporosis in children [64].

No differences in terms of BMD accumulation, suppression of bone turnover markers or incidence of fractures have been reported when comparing pamidronate with alendronate in children with osteogenesis imperfecta [2,65].

Table 4 reports contraindications, doses and dosing intervals for the most commonly used BPs in pediatrics. Current references for administration, duration of treatment and long-term safety of BP treatment require further investigation.

Osteoporosis secondary to chronic inflammation in leukemia patients may also require treatment with BPs [29,30].

In our experience, markers of apposition (bone ALP, osteocalcin, N-terminal propeptide of procollagen type 1-P1NP, etc.) can be checked every 6 months, together with the levels of PTH and 25 OH vitamin D (systematically supplemented to maintain levels > 30 ng/mL). DXA is performed every 12 months to evaluate changes in BMD during treatment.

Although BPs are still the most commonly used drug to increase BMD, improve mobility and reduce pain and fracture recurrence, promising new therapies are being developed for specific pediatric conditions.

Denosumab (DB) is a potent anti-bone resorption drug. It is a humanized monoclonal antibody against RANKL, to which it binds with high affinity and specificity. By binding to and blocking RANKL, DB reduces osteoclast formation and activity. Clinical studies have shown that DB has a similar safety profile to BPs and may be equally effective in the treatment of OI, rheumatoid arthritis and bone metastases in adults. DB has a shorter half-life than BPs; however, upon discontinuation, it presents a “rebound effect”, i.e., an increase in vertebral fractures after discontinuation of treatment, and severe hypercalcemia is observed within a few weeks of administration. Given these considerations, DB is currently recommended in childhood under limited, specific conditions, such as the presence of giant cell tumors, OI type 6 and in cases of poor response to bisphosphonates in juvenile Paget’s disease. In some countries, such as Italy, its use is off-label in children [2,66,67,68,69].

Finally, teriparatide, an analog of the parathyroid hormone, is under study thanks to its powerful osteoblast-stimulating activity; only short-term studies have been published to date, mainly in limited series of patients with specific conditions such as Duchenne muscular dystrophy [68,69,70,71] and autosomal dominant hypocalcemia type 1 [72]. Therefore, it is far too early to draw any conclusions.

There is growing interest in the potential use of extracellular vesicles as therapeutic entities, particularly for stem cell approaches. Extracellular vesicles are particles secreted by cells outside the cell and are enveloped in a double layer of phospholipids that play an important role in various biological processes and diseases, including osteoporosis. It has been shown that senescent endothelial cells secrete microvesicles that inhibit the osteogenic differentiation of mesenchymal stem cells [73].

Finally, children with “reduced-use” osteoporosis, such as those with muscle paralysis, should perform passive range-of-motion exercises and assisted physical exercises to improve muscle strength and coordination, as it has been clearly shown that these procedures can lead to an improvement in BMD [74,75].

## 6. Conclusions

Increasing awareness of the various forms and risks of pediatric osteoporosis and referral to a specialist team for appropriate management can lead to early detection and treatment of asymptomatic fractures and prevention of further bone damage.

Osteoporosis and bone fragility in children are caused by different factors. Currently, environmental factors, including pollutants, must be considered.

New skeletal imaging techniques are of great help in evaluating BMD in childhood. In particular, REMS, thanks to its radiation-free approach, could be used soon for mass population screening, for prevention programs and in the diagnostic and therapeutic follow-up of bone fragility in patients of pediatric age.

Prevention programs, early diagnosis and personalized therapy are important. An osteoporotic child should be approached by a multidisciplinary team, including a pediatric endocrinologist, a radiologist, an orthopedic surgeon, a physiotherapist and a dietician. The goal is to maximize bone mass and improving bone health, which has lifelong implications and effects. The development of new drugs capable of restoring physiological anabolic bone activity in children with osteoporosis is on the way.

## Figures and Tables

**Table 1:** Osteogenesis imperfecta classification [6,8].

Nomenclature	Gene	Clinical Classification by Severity Grading Scale	
Non-deforming OI with blue sclerae	1. *COL1A1* 2. *COL1A2*	MILD OI: Bone problems - Prenatally: Rarely specific US features at 20 weeks of pregnancy No intra-uterine fractures of the long bones or postpartum bowing - Postnatally: Rare congenital fractures Straight long bones without intrinsic deformities Minimal vertebral crush fractures Annual number of fractures: 1 or fewer than 1	Bone density and other features: - In the postnatal period: Normal or quite normal growth rate and height Normally walking children, except in times of acute fracture No chronic bone pain or pain controlled with medications Blue sclerae Late-onset hearing loss Dentinogenesis imperfecta
Common Variable with normal sclerae	1. *COL1A1* 2. *COL1A2* 3. *WNT1*a 1. *CRTAP* 2. *PPIB* 3. *SP7* 1. *PLS3*	MODERATE OI: Bone problems: - Prenatally: specific US features at 20 weeks of pregnancy Rarely, fetal fractures or bowing of long bones (may increase in the last trimester) - Postnatally: no change with bisphosphonate therapy Occasionally congenital fractures Anterior bowing of legs and thighs Bowing of long bones related to immobilization due to recurrent fractures Vertebral crush fractures More than 1 annual prepubertal fractures (on average, 3 within a wide range)	Bone density and other features: - In the postnatal period: Reduced growth rate and height Bone mineral density of the lumbar spine: Z score >−2.5 <−1.5 with a wide range of variability
OI with interosseous membranes Calcifications	1. *IFITM5*		
Progressively deforming	1. *COL1A1* 2. *COL1A2* 1. *BMP1* 2. *CRTAP* 3. *FKBP10* 4. *LEPRE1* 5. *PLOD2* 6. *PPIB* 7. *SERPINF1* 8. *SERPINH1* 9. *MEM38B* 10. *WNT1* 11. *CREB3L1*	SEVERE OI: Bone problems: - Prenatally: specific US features at 20 weeks of pregnancy: Shortening of long bones Fractures or long-bone incurvation with a certain “under-modeling” Thin ribs with absent or discontinuous rib fractures (intermediate cases between severe and extremely severe OI have few rib fractures but crumpled long bones) - Postnatally: Progressive deformities of the long bones and spine (unrelated to fractures) Multiple vertebral fractures due to crushing More than 3 fractures/year	Bone density and other features: - Prenatal period: Reduced bone mineralization - Postnatal period: Reduced mineralization (unmodified by bisphosphonate therapy) Marked impairment of growth rate Wheelchair dependence Bone mineral density of the lumbar spine: Z score usually <−3.0 (wide range in relation to age, given that the measurement is height-dependent) Chronic pain unless treated with bisphosphonates
Letal perinatal OI	1. *COL1A1* 2. *COL1A2* 1. *CRTAP* 2. *LEPRE1* 3. *PPIB*	EXTREMELY SEVERE OI: Bone problems: - Prenatally: Detection of US features at 20 weeks of pregnancy: Shortness of long bones Fractures and/or curvature of the long bones, with severe “under-modeling”, which leads to crumpling (accordion-like) of the long bones Thick, continuous beaded ribs due to the presence of multiple fracture sites on thin ribs (formerly described as OI types 2-A and 2-B) - Postnatal: Multiple fractures of the long bones and ribs All vertebrae are hypoplastic and crushed	Bone density and other features: - Prenatal period: Reduced postnatal mineralization - Postnatal period: Thighs held in fixed abduction and external rotation, with limited movement of most joints Clinical indicators of severe chronic pain (paleness, sweating, moaning or grimacing upon passive movements) Reduced skull ossification Small chest Shortened and compacted femurs with an accordion appearance Respiratory distress leading to perinatal mortality Lethal perinatal course

**Table 2:** List of the most relevant imaging modalities for the assessment of bone health in patients of pediatric age.

MAIN IMAGING MODALITIES FOR BONE HEALTH EVALUATION IN CHILDREN			
Imaging Modality	Sites of Application	Relevant Advantages	Relevant Disadvantages
DXA	Lumbar spine Total body less head Radius Hip (not recommended)	Gold standard	Children < 4 years of age Radiation
CT Includes QCT, pQCT (and HR-pQCT) and vQCT	Radius (pQCT) Tibia (pQCT) Femur (pQCT)	Three-dimensional study Cortical vs. trabecular bone	Radiation
QUS	Heel Proximal phalanges of the hand Tibia Radius	No radiation Easy and fast to use Cost-effective Portable devices	Lack of solid reference values Not applicable to axial skeleton
MRI Includes micro-MRI		Volumetric measurements Cortical vs. trabecular bone	Long image acquisition time Sedation usually required
REMS	Lumbar vertebrae Proximal femur	No radiation Fast to use	Lack of solid reference values Still confined to research setting

DXA: dual-energy X-ray absorptiometry; CT: computed tomography; QCT: quantitative computed tomography; pQCT: peripheral quantitative computed tomography; vQCT: vertebral quantitative computed tomography; QUS: quantitative ultrasound; MRI: magnetic resonance imaging; REMS: radiofrequency echographic multi-spectrometry.

**Table 3:** Daily calcium and vitamin D requirements according to age.

Age	Calcium (mg)	25 OH Vitamin D (IU)
0–6 months	200	400
6–12 months	260	400
1–3 years	700	600
4–8 years	1000	600
9–18 years	1300	600

**Table 4:** Doses, contraindications and dosing intervals for the most commonly used bisphosphonates (BPs) in children and adolescents.

Drug	Contraindications	Administration	Dose
Pamidronate (2nd generation)	Hypocalcemia, severe renal failure, hypersensitivity	IV, diluted in 100–250 mL physiological saline solution for 3–4 h	<1 year: 0.5 mg/kg every 2 months 1–2 years: 0.25–0.5 mg/kg/day for 3 days every 3 months 2–3 years: 0.375–0.75 mg/kg/day for 3 days every 3 months >3 years: 0.5–1 mg/kg/day for 3 days every 4 months Maximum dose: 60 mg/dose and 11.5 mg/kg/year
Neridronate (3rd generation)	Hypocalcemia, severe renal failure, hypersensitivity	IV, diluted in 200–250 mL physiological saline solution for 3 h	1–2 mg/kg every 3–4 months
Zolendronate (3rd generation)	Hypocalcemia, severe renal failure, hypersensitivity	IV, diluted in 50 mL physiological saline solution for 30−45 min	0.0125–0.05 mg/kg every 6–12 months (maximum dose: 4 mg)
Alendronate (2nd generation)	Hypocalcemia, delayed esophageal emptying, severe renal failure, hypersensitivity, inability to stand or sit for at least 30 min	Oral	1–2 mg/kg/week <40 kg: 5 mg/day or 35 mg/week >40 kg: 10 mg/day or 70 mg/week Maximum dose: 70 mg/week
Risendronate (3rd generation)	Hypocalcemia, delayed esophageal emptying, severe renal failure, hypersensitivity, inability to stand or sit for at least 30 min	Oral	15 mg/week (<40 kg); 30 mg/week (>40 kg) Maximum dose: 30 mg/week

## Data Availability

Not applicable.

## Abbreviations

BMD	bone mineral density
DXA	dual-energy X-ray absorptiometry
OI	osteogenesis imperfecta
EDCs	endocrine-disrupting chemicals
QCT	quantitative computed tomography
QUS	quantitative ultrasound
REMS	radiofrequency echographic multi-spectrometry
MRI	magnetic resonance imaging
BPs	bisphosphonates
DB	denosumab

## References

[1] Ciancia S., van Rijn R.R., Högler W., Appelman-Dijkstra N.M., Boot A.M., Sas T.C.J., Renes J.S. (2022). Osteoporosis in children and adolescents: When to suspect and how to diagnose it. Eur. J. Pediatr..

[2] Płudowski P., Kos-Kudła B., Walczak M., Fal A., Zozulińska-Ziółkiewicz D., Sieroszewski P., Peregud-Pogorzelski J., Lauterbach R., Targowski T., Lewiński A. (2023). Guidelines for Preventing and Treating Vitamin D Deficiency: A 2023 Update in Poland. Nutrients.

[3] Gordon C.M., Leonard M.B., Zemel B.S., International Society for Clinical Densitometry (2014). 2013 Pediatric Position Development Conference: Executive summary and reflections. J. Clin. Densitom..

[4] Galindo-Zavala R., Bou-Torrent R., Magallares-López B., Mir-Perelló C., Palmou-Fontana N., Sevilla-Pérez B., Medrano-San Ildefonso M., González-Fernández M.I., Román-Pascual A., Alcañiz-Rodríguez P. (2020). Expert panel consensus recommendations for diagnosis and treatment of secondary osteoporosis in children. Pediatr. Rheumatol. Online J..

[5] Bakirhan H., Karabudak E. (2023). Effects of inulin on calcium metabolism and bone health. Int. J. Vitam. Nutr. Res..

[6] Unger S., Ferreira C.R., Mortier G.R., Ali H., Bertola D.R., Calder A., Cohn D.H., Cormier-Daire V., Girisha K.M., Hall C. (2023). Nosology of genetic skeletal disorders: 2023 revision. Am. J. Med. Genet. Part A.

[7] Kang H., Aryal A.C.S., Marini J.C. (2017). Osteogenesis imperfecta: New genes reveal novel mechanisms in bone dysplasia. Transl. Res..

[8] Van Dijk F.S., Pals G., Van Rijn R.R., Nikkels P.G., Cobben J.M. (2010). Classification of Osteogenesis Imperfecta revisited. Eur. J. Med. Genet..

[9] Van Dijk F.S., Sillence D.O. (2014). Osteogenesis imperfecta: Clinical diagnosis, nomenclature and severity assessment. Am. J. Med. Genet. Part A.

[10] Ohata Y., Kitaoka T., Ishimi T., Yamada C., Nakano Y., Yamamoto K., Takeyari S., Nakayama H., Fujiwara M., Kubota T. (2023). Association of trabecular bone score and bone mineral apparent density with the severity of bone fragility in children and adolescents with osteogenesis imperfecta: A cross-sectional study. PLoS ONE.

[11] Van Dijk F.S., Byers P.H., Dalgleish R., Malfait F., Maugeri A., Rohrbach M., Symoens S., Sistermans E.A., Pals G. (2012). EMQN best practice guidelines for the laboratory diagnosis of osteogenesis imperfecta. Eur. J. Hum. Genet..

[12] Deguchi M., Tsuji S., Katsura D., Kasahara K., Kimura F., Murakami T. (2021). Current Overview of Osteogenesis Imperfecta. Medicina.

[13] Cannalire G., Pilloni S., Esposito S., Biasucci G., Di Franco A., Street M.E. (2023). Alkaline phosphatase in clinical practice in childhood: Focus on rickets. Front. Endocrinol..

[14] Saito M., Marumo K. (2018). The Effects of Homocysteine on the Skeleton. Curr. Osteoporos. Rep..

[15] Alkaissi H., McFarlane S.I. (2023). Hyperhomocysteinemia and Accelerated Aging: The Pathogenic Role of Increased Homocysteine in Atherosclerosis, Osteoporosis, and Neurodegeneration. Cureus.

[16] Herrmann M., Widmann T., Herrmann W. (2005). Homocysteine--a newly recognised risk factor for osteoporosis. Clin. Chem. Lab. Med..

[17] Baron R., Kneissel M. (2013). WNT signaling in bone homeostasis and disease: From human mutations to treatments. Nat. Med..

[18] Biha N., Ghaber S.M., Hacen M.M., Collet C. (2016). Osteoporosis-Pseudoglioma in a Mauritanian Child due to a Novel Mutation in LRP5. Case Rep. Genet..

[19] Robinson M.E., Rauch F. (2019). Mendelian bone fragility disorders. Bone.

[20] Trifirò G., Marelli S., Viecca M., Mora S., Pini A. (2015). Areal bone mineral density in children and adolescents with Marfan syndrome: Evidence of an evolving problem. Bone..

[21] Basalom S., Rauch F. (2020). Bone Disease in Patients with Ehlers-Danlos Syndromes. Curr. Osteoporos. Rep..

[22] Whyte M.P. (2016). Hypophosphatasia—Aetiology, nosology, pathogenesis, diagnosis and treatment. Nat. Rev. Endocrinol..

[23] Franceschi R., Vincenzi M., Camilot M., Antoniazzi F., Freemont A.J., Adams J.E., Laine C., Makitie O., Mughal M.Z. (2015). Idiopathic juvenile osteoporosis: Clinical experience from a single centre and screening of LRP5 and LRP6 genes. Calcif. Tissue Int..

[24] Rouleau C., Malorie M., Collet C., Porquet-Bordes V., Gennero I., Eddiry S., Laroche M., Salles J.P., Couture G., Edouard T. (2022). Diagnostic yield of bone fragility gene panel sequencing in children and young adults referred for idiopathic primary osteoporosis at a single regional reference centre. Bone Rep..

[25] Mora S. (2008). Celiac disease in children: Impact on bone health. Rev. Endocr. Metab. Disord..

[26] Lungaro L., Manza F., Costanzini A., Barbalinardo M., Gentili D., Caputo F., Guarino M., Zoli G., Volta U., De Giorgio R. (2023). Osteoporosis and Celiac Disease: Updates and Hidden Pitfalls. Nutrients.

[27] Zacay G., Weintraub I., Regev R., Modan-Moses D., Levy-Shraga Y. (2023). Fracture risk among children and adolescents with celiac disease: A nationwide cohort study. Pediatr. Res..

[28] Benchimol E.I., Ward L.M., Gallagher J.C., Rauch F., Barrowman N., Warren J., Beedle S., Mack D.R. (2007). Effect of calcium and vitamin D supplementation on bone mineral density in children with inflammatory bowel disease. J. Pediatr. Gastroenterol. Nutr..

[29] Mostoufi-Moab S., Ward L.M. (2019). Skeletal Morbidity in Children and Adolescents during and following Cancer Therapy. Horm. Res. Paediatr..

[30] Verwaaijen E.J., Ma J., de Groot-Kruseman H.A., Pieters R., van der Sluis I.M., van Atteveld J.E., Halton J., Fernandez C.V., Hartman A., de Jonge R. (2021). DCOG-ALL9 and Canadian STOPP Consortia. A Validated Risk Prediction Model for Bone Fragility in Children with Acute Lymphoblastic Leukemia. J. Bone Min. Res..

[31] Verrotti A., Coppola G., Parisi P., Mohn A., Chiarelli F. (2010). Bone and calcium metabolism and antiepileptic drugs. Clin. Neurol. Neurosurg..

[32] Misra M. (2012). Effects of hypogonadism on bone metabolism in female adolescents and young adults. Nat. Rev. Endocrinol..

[33] Kenkre J.S., Bassett J. (2018). The bone remodelling cycle. Ann. Clin. Biochem..

[34] Williams G.R., Bassett J.H.D. (2018). Thyroid diseases and bone health. J. Endocrinol. Invest..

[35] Sakka S.D. (2022). Osteoporosis in children and young adults. Best Pract. Res. Clin. Rheumatol..

[36] Carson J.A., Manolagas S.C. (2015). Effects of sex steroids on bones and muscles: Similarities, parallels, and putative interactions in health and disease. Bone.

[37] Ward L.M. (2020). Glucocorticoid-Induced Osteoporosis: Why Kids Are Different. Front. Endocrinol.

[38] Cohen A., Shane E. (2003). Osteoporosis after solid organ and bone marrow transplantation. Osteoporos. Int..

[39] Fleishman N., Richardson T., Attard T. (2020). The Clinical Characteristics of Fractures in Pediatric Patients Exposed to Proton Pump Inhibitors. J. Pediatr. Gastroenterol. Nutr..

[40] Zhang Y., Zheng Y.X., Zhu J.M., Zhang J.M., Zheng Z. (2015). Effects of antiepileptic drugs on bone mineral density and bone metabolism in children: A meta-analysis. J. Zhejiang Univ. Sci. B.

[41] LaBrie J.W., Boyle S., Earle A., Almstedt H.C. (2018). Heavy Episodic Drinking Is Associated with Poorer Bone Health in Adolescent and Young Adult Women. J. Stud. Alcohol Drugs.

[42] Dorn L.D., Pabst S., Sontag L.M., Kalkwarf H.J., Hillman J.B., Susman E.J. (2011). Bone mass, depressive, and anxiety symptoms in adolescent girls: Variation by smoking and alcohol use. J. Adolesc. Health.

[43] Perrone S., Caporilli C., Grassi F., Ferrocino M., Biagi E., Dell’Orto V., Beretta V., Petrolini C., Gambini L., Street M.E. (2023). Prenatal and Neonatal Bone Health: Updated Review on Early Identification of Newborns at High Risk for Osteopenia. Nutrients.

[44] Buckley J.P., Kuiper J.R., Lanphear B.P., Calafat A.M., Cecil K.M., Chen A., Xu Y., Yolton K., Kalkwarf H.J., Braun J.M. (2021). Associations of Maternal Serum Perfluoroalkyl Substances Concentrations with Early Adolescent Bone Mineral Content and Density: The Health Outcomes and Measures of the Environment (HOME) Study. Environ. Health Perspect..

[45] Shulhai A.M., Palanza P., Street M.E. (2023). Current Evidence on the Effects of Endocrine-Disrupting Chemicals (EDCs) on Bone Growth and Health. Expo Health.

[46] Formosa M.M., Christou M.A., Mäkitie O. (2024). Bone fragility and osteoporosis in children and young adults. J. Endocrinol. Invest..

[47] Vasikaran S., Eastell R., Bruyère O., Foldes A.J., Garnero P., Griesmacher A., McClung M., Morris H.A., Silverman S., Trenti T. (2011). IOF-IFCC Bone Marker Standards Working Group. Markers of bone turnover for the prediction of fracture risk and monitoring of osteoporosis treatment: A need for international reference standards. Osteoporos. Int..

[48] Bachrach L.K., Gordon C.M., Section on Endocrinology (2016). Bone Densitometry in Children and Adolescents. Pediatrics.

[49] Crabtree N.J., Arabi A., Bachrach L.K., Fewtrell M., El-Hajj Fuleihan G., Kecskemethy H.H., Jaworski M., Gordon C.M., International Society for Clinical Densitometry (2014). Dual-energy X-ray absorptiometry interpretation and reporting in children and adolescents: The revised 2013 ISCD Pediatric Official Positions. J. Clin. Densitom..

[50] Madhuchani D., Seneviratne S.N., Ward L.M. (2023). Bone health in childhood and adolescence: An overview on dual-energy X-ray absorptiometry scanning, fracture surveillance and bisphosphonate therapy for low-middle-income countries. Front. Endocrinol..

[51] Kindler J.M., Lappe J.M., Gilsanz V., Oberfield S., Shepherd J.A., Kelly A., Winer K.K., Kalkwarf H.J., Zemel B.S. (2019). Lumbar Spine Bone Mineral Apparent Density in Children: Results from the Bone Mineral Density in Childhood Study. J. Clin. Endocrinol. Metab..

[52] Di Iorgi N., Maruca K., Patti G., Mora S. (2018). Update on bone density measurements and their interpretation in children and adolescents. Best. Pr. Res. Clin. Endocrinol. Metab..

[53] Raum K., Grimal Q., Varga P., Barkmann R., Glüer C.C., Laugier P. (2014). Ultrasound to assess bone quality. Curr. Osteoporos. Rep..

[54] Casciaro S., Peccarisi M., Pisani P., Franchini R., Greco A., De Marco T., Grimaldi A., Quarta L., Quarta E., Muratore M. (2016). An Advanced Quantitative Echosound Methodology for Femoral Neck Densitometry. Ultrasound Med. Biol..

[55] Engelke K. (2017). Quantitative Computed Tomography-Current Status and New Developments. J. Clin. Densitom..

[56] Zemel B.S. (2011). Quantitative computed tomography and computed tomography in children. Curr. Osteoporos. Rep..

[57] Jaworski M., Kobylińska M., Graff K. (2021). Peripheral quantitative computed tomography of the lower leg in children and adolescents: Bone densities, cross-sectional sizes and muscle distribution reference data. J. Musculoskelet. Neuronal Interact..

[58] Modlesky C.M., Whitney D.G., Carter P.T., Allerton B.M., Kirby J.T., Miller F. (2014). The pattern of trabecular bone microarchitecture in the distal femur of typically developing children and its effect on processing of magnetic resonance images. Bone.

[59] Baroncelli G.I. (2008). Quantitative ultrasound methods to assess bone mineral status in children: Technical characteristics, performance, and clinical application. Pediatr. Res..

[60] Shalof H., Dimitri P., Shuweihdi F., Offiah A.C. (2021). Which skeletal imaging modality is best for assessing bone health in children and young adults compared to DXA? A systematic review and meta-analysis. Bone.

[61] Gazzotti S., Aparisi Gómez M.P., Schileo E., Taddei F., Sangiorgi L., Fusaro M., Miceli M., Guglielmi G., Bazzocchi A. (2023). High-resolution peripheral quantitative computed tomography: Research or clinical practice?. Br. J. Radiol..

[62] Chalcraft J.R., Cardinal L.M., Wechsler P.J., Hollis B.W., Gerow K.G., Alexander B.M., Keith J.F., Larson-Meyer D.E. (2020). Vitamin D Synthesis Following a Single Bout of Sun Exposure in Older and Younger Men and Women. Nutrients.

[63] Ciancia S., Högler W., Sakkers R.J.B., Appelman-Dijkstra N.M., Boot A.M., Sas T.C.J., Renes J.S. (2023). Osteoporosis in children and adolescents: How to treat and monitor?. Eur. J. Pediatr..

[64] Ward L.M., Choudhury A., Alos N., Cabral D.A., Rodd C., Sbrocchi A.M., Taback S., Padidela R., Shaw N.J., Hosszu E. (2021). Zoledronic Acid vs. Placebo in Pediatric Glucocorticoid-induced Osteoporosis: A Randomized, Double-blind, Phase 3 Trial. J. Clin. Endocrinol. Metab..

[65] Zhao H., Ding Y., Yang J., Luo Y., Xu Z., Miao J. (2022). Efficacy and safety of bisphosphonates on childhood osteoporosis secondary to chronic illness or its treatment: A meta-analysis. Ther. Adv. Chronic. Dis..

[66] Biasucci G., Donini V., Cannalire G. (2024). Rickets Types and Treatment with Vitamin D and Analogues. Nutrients.

[67] Kawahara M., Kuroshima S., Sawase T. (2021). Clinical considerations for medication-related osteonecrosis of the jaw: A comprehensive literature review. Int. J. Implant Dent..

[68] Pang K.L., Low N.Y., Chin K.Y. (2020). A Review on the Role of Denosumab in Fracture Prevention. Drug Des. Devel. Ther..

[69] Wang D., Tang X., Shi Q., Wang R., Ji T., Tang X., Guo W. (2023). Denosumab in pediatric bone disorders and the role of RANKL blockade: A narrative review. Transl. Pediatr..

[70] Kendler D.L., Cosman F., Stad R.K., Ferrari S. (2022). Denosumab in the Treatment of Osteoporosis: 10 Years Later: A Narrative Review. Adv. Ther..

[71] Nasomyont N., Keefe C., Tian C., Hornung L., Khoury J., Tilden J.C., Hochwalt P., Jackson E., Rybalsky I., Wong B.L. (2020). Safety and efficacy of teriparatide treatment for severe osteoporosis in patients with Duchenne muscular dystrophy. Osteoporos. Int..

[72] Hawkes C.P., Shulman D.I., Levine M.A. (2020). Recombinant human parathyroid hormone (1-84) is effective in CASR-associated hypoparathyroidism. Eur. J. Endocrinol..

[73] Fang F., Yang J., Wang J., Li T., Wang E., Zhang D., Liu X., Zhou C. (2024). The role and applications of extracellular vesicles in osteoporosis. Bone Res..

[74] Antoniazzi F., Monti E., Venturi G., Franceschi R., Doro F., Gatti D., Zamboni G., Tatò L. (2010). GH in combination with bisphosphonate treatment in osteogenesis imperfecta. Eur. J. Endocrinol..

[75] Głuszko P., Sewerynek E., Misiorowski W., Konstantynowicz J., Marcinowska-Suchowierska E., Blicharski T., Jabłoński M., Franek E., Kostka T., Jaworski M. (2023). Guidelines for the diagnosis and management of osteoporosis in Poland. Update 2022. Endokrynol. Pol..

